# Targeting Myotonic Dystrophy Type 1 with Metformin

**DOI:** 10.3390/ijms23052901

**Published:** 2022-03-07

**Authors:** Mikel García-Puga, Ander Saenz-Antoñanzas, Ander Matheu, Adolfo López de Munain

**Affiliations:** 1 Neuromuscular Diseases Group, Biodonostia Health Research Institute, 20014 San Sebastian, Spain; mikel.garcia@biodonostia.org; 2 Cellular Oncology Group, Biodonostia Health Research Institute, 20014 San Sebastian, Spain; ander.saenz@biodonostia.org; 3 Network Center for Biomedical Research in Neurodegenerative Diseases (CIBERNED-CIBER), Carlos III Institute, 28031 Madrid, Spain; 4 Basque Foundation for Science (IKERBASQUE), 48009 Bilbao, Spain; 5 Centro de Investigación Biomédica en Red Fragilidad y Envejecimiento Saludable (CIBERfes), Carlos III Institute, 28029 Madrid, Spain; 6 Neurology Department, Donostia University Hospital, OSAKIDETZA, 20014 San Sebastian, Spain; 7 Department of Neurosciences, Faculty of Medicine and Nursery, University of the Basque Country, 20014 San Sebastian, Spain

**Keywords:** myotonic dystrophy, metformin, therapeutic strategies, aging

## Abstract

Myotonic dystrophy type 1 (DM1) is a multisystemic disorder of genetic origin. Progressive muscular weakness, atrophy and myotonia are its most prominent neuromuscular features, while additional clinical manifestations in multiple organs are also common. Overall, DM1 features resemble accelerated aging. There is currently no cure or specific treatment for myotonic dystrophy patients. However, in recent years a great effort has been made to identify potential new therapeutic strategies for DM1 patients. Metformin is a biguanide antidiabetic drug, with potential to delay aging at cellular and organismal levels. In DM1, different studies revealed that metformin rescues multiple phenotypes of the disease. This review provides an overview of recent findings describing metformin as a novel therapy to combat DM1 and their link with aging.

## 1. Myotonic Dystrophy Type 1 (DM1)

Myotonic dystrophies (DM) are dominantly inherited, multisystemic diseases that share the core features of myotonia, muscle weakness, muscular dystrophy, early onset cataracts, cardiac conduction defects, endocrine disorders and increased risk of developing neoplasias [1,2]. DM is classified into two distinct forms based on clinical and molecular characteristics: DM type 1 (dystrophia myotonica type I; DM1; OMIM# 160900, also known as Steinert’s disease) and DM type 2 (dystrophia myotonica type 2, DM2; OMIM #602668). Hans Steinert was the first to describe DM over a century ago. Although, the identification of the molecular cause responsible for DM1 came 90 years later. In 1992, it was reported that DM1 is caused by an unstable expansion of CTG (cytosine-thymine-guanine) trinucleotide repeat in the 3′ noncoding region of the *Dystrophia Myotonic Protein Kinase* gene (*DMPK*) [3,4], whereas DM2 present an unstable expansion of a tetra-nucleotide CCTG (cytosine-cytosine-thymine-guanine) repeat in the first intron of *Cellular Nucleic Acid Binding Protein* gene (*CNBP*, often-termed zinc finger 9 gene -*ZNF9*-), [5,6].

Both diseases are characterized by altered splicing of several downstream effector genes with negative effects on multiple tissues, leading to complex clinical manifestations. DM patients suffer a broad variety of symptoms affecting the three muscle types: cardiac, skeletal and smooth muscles. DM1 affects mainly distal muscles and shows a prominent loss of type 1 fibers whereas DM2 affects proximal muscles and type 2 fibers [7,8,9]. Moreover, patients with DM, present a multisystem degenerative process [10]. DM1 is more common and represents a more severe phenotype than DM2. Indeed, DM1 is the most common form of adult-onset muscular dystrophy affecting 1 out of 8000 (or 12.5/100,000) people worldwide, with a high prevalence in some specific areas such as Quebec (Canada) and the Basque Country (Spain) [11,12]. However, recent studies have shown that the prevalence is underestimated, estimating a prevalence of 48/100,000 [13].

In DM1, the length of CTG expansion is associated with the age of onset of the disease and its severity. In this sense, mildly affected persons have 50 to 100 repeats, patients with classic DM1 have 100 to 1000 repeats, and those with onset at birth can have more than 2000 repeats. In addition, the length of the expansion increases over time [14]. In recent years, interruptions at the 5′ and 3′ ends of the CTG expansion of pathological *DMPK* transcripts have been described in around 3–5% of DM1 patients [15,16]. These sequences are mainly composed of unstable CCG, CGG, CTC and CAG interruptions, and they have been associated as a mechanism that increases phenotypical variability, although further studies to characterize their impact are needed. Based on the manifestation of the main symptoms, the repeat length and the age of onset, four clinical subtypes of DM1 are recognized: congenital (CDM), pediatric, adult, and late-onset DM1 [17,18,19].

The pathogenic mechanism that leads to DM1 was first associated with DMPK haploinsufficiency due to the inability of the protein to complete its final location and function [20,21,22,23]. Abnormal expansion of CTG repeats in DMPK disrupts the chromatin structure and affects the expression of neighboring genes such as the homeodomain-encoding transcription factor *SIX5* and WD repeat-containing protein (*DMWD*) gene [8]. Mutant *DMPK* transcripts containing CTG expansions lead to formation of transcript aggregates that accumulate in the nucleus in ribonuclear foci [24]. Those aggregates interfere with proteins that play an important role in RNA metabolism, including members of muscleblind-like (MBNL) and CUGBP Elav-like family (CELF) proteins that are implicated in the regulation of alternative splicing [25,26]. Alteration of these proteins lead to the accumulation of fetal alternative splicing isoforms of transcripts in adult tissues [27,28]. In addition, a potential role of methylation has been described in congenital DM1, associated with larger maternal CTG expansions [29].

## 2. Myotonic Dystrophy and Aging: Link at Clinical Level

Aging is characterized by a progressive loss of physiological function, which drives the development of chronic morbidities including metabolic, cardiovascular, neurodegenerative disorders, cancer, as well as geriatric symptoms like frailty and dependency. Patients with DM1 present a multisystem degenerative process that resembles several of these aging alterations.

DM1 patients suffer a broad variety of symptoms that affect the three muscle types. It should be noted that the most affected tissues in these patients are those that present a greater increase in the length of the CTG, such as muscle tissue [30]. Cardiac failure is common in DM1 patients, often manifested as arrhythmias and conduction defects [31,32]. The frequency of heart failure correlates with age, male gender, length of the tandem repeat and the degree of neuromuscular disability [31,32]. Indeed, cardiac disease is responsible for 30% of deaths in patients with myotonic dystrophy. Symptoms involving the smooth muscle, such as dysphagia, constipation, intestinal pseudo-obstruction and diarrhea, are also relatively frequent in DM1 patients [10]. Interestingly, the histology of DM1 skeletal muscles resembles that of aged muscles, with fiber size variability, centrally located nuclei with chromatin clumps and fiber atrophy. Muscle regeneration also seems to be decreased, probably due to satellite cell dysfunction, which may fail to activate and/or differentiate to muscle upon myogenic stimuli [33,34].

The CNS is also affected in DM1 patients. In particular, congenital and childhood-onset DM1 patients suffer mental retardation, whereas patients with the adult-onset forms may show varying degrees of cognitive dysfunction, where a positive correlation between triplet expansion length and patients’ age is observed. Nowadays, there are data supporting an age-dependent progressive cognitive decline in DM1 patients [35], that correlates with brain atrophy [36].

DM1 patients show insulin resistance due to the aberrant splicing of the insulin receptor (IR) mRNA, which is highly expressed in skeletal muscle. Consequently, patients present a reduced responsiveness to insulin as compared to healthy individuals [37,38]. DM1 patients also show several metabolic defects that are also common in aged individuals, such as glucose resistance, hyperinsulinemia and the development of diabetes mellitus [37,39]. Some of these dysfunctions seem to correlate with the length of the repeat expansion [40]. On the other hand, respiratory difficulties are common in DM1 and are one of the main causes of death in these patients [17]. It is also the main factor that influences the deterioration of quality of life [8]. DM1 patients also present hepatic deterioration. Indeed, 66% of patients show abnormal hepatic enzyme levels and non-alcoholic steatosis [8,41]. Ocular complications, including ptosis, weakness of the ocular muscle and cataracts are also common in DM1 patients, and other less frequent features, such as retinal changes or macular degeneration, may also be present [42]. DM1 patients may also suffer fertility dysfunction. Approximately two thirds of affected males have reduced sperm quality as a result of testicular atrophy [43,44]. Affected female fertility is less well documented, but there may be a higher incidence of infertility, spontaneous abortions and, in rare cases, premature ovarian failure [45].

Recent studies have provided evidence that DM1 patients are at higher risk of developing different types of cancers [2,46,47,48,49,50]. However, the risk factors and molecular mechanisms of DM1-carcinogenesis are largely unexplored. Possibilities to explain this come from genetic predisposition to cancer that is driven by specific aspects of DM1 pathophysiology [51] and by the alteration of cancer-related pathways [46].

As a consequence of multisystem deterioration, patients with DM1 have a reduced life expectancy with a mean age at death of 53 years and a mortality rate approximately 7.3 times higher than of an age-matched general population. The cause of death is respiratory failure in approximately 40% of cases and cardiac in approximately 30% of cases [17,52,53]. In addition, the increased risk of developing cancer in DM1 patients, represents the third leading cause of death [46,47]. Thus, DM1 patients present multiple clinical phenotypes that resembles an accelerated aging process [54,55].

## 3. Myotonic Dystrophy and Aging: Link at Molecular Level

Deregulation of several hallmarks of aging [56], including stem cell exhaustion, cellular senescence, telomere attrition, mitochondrial dysfunction and deregulation of nutrient sensing have been associated with the pathophysiology of DM1 [54,55].

In regard to stem cell activity, the number of satellite cells in a DM1 mouse model is lower than wild-type controls [57]. In line, human DM1 satellite cells showed a considerably lower proliferative rate than age-matched controls. They also showed accumulation of senescence markers such as increased senescence-associated beta galactosidase, high levels of cyclin D1 and hypophosphorylated Rb [58,59,60]. Moreover, DM1 myoblasts [61,62,63] and neural stem cells [64] have impaired cell proliferation [61,62,64]. The myogenic program is also compromised in these cells [65]. There is a general agreement that the impediment of terminal differentiation is a feature of myoblasts derived from skeletal muscles from DM1 patients who carry long expanded CTG repeats [59,61,63,65]. In addition, there is a defective differentiation and maturation of DM1 myogenic progenitors in vitro, resulting in smaller and thinner myotubes, with a 30% lower fusion index and the lack of expression of mature myosin forms [62]. This lack of fiber maturation has also been confirmed in DM1 muscle biopsies, where late myogenic differentiation markers are not fully expressed [66]. Moreover, DM1 myoblasts seem to have impaired cell cycle withdrawal, probably due to the inability to induce the expression of p21 [61]. These abnormalities in skeletal muscle myogenesis and premature senescence of satellite cells resemble physiological satellite cell aging. Additional senescence features, such as cytoplasmic vacuolization, accumulation of heterochromatin and impaired pre-mRNA maturation have also been found in DM1 cells [33,34], although their function in the pathophysiology of the disease remains poorly described.

In regard to telomere length, different studies tried to connect the telomere length to DM1 phenotypes. Surprisingly, studies in human cells in vitro and in vivo did not find differences in telomere length between controls and DM1 cells. However, DM1 satellite cells in vitro and PBMCs in vivo display an exacerbated telomere shortening rate [58,59,67], further linking DM1 to premature aging.

Concerning mitochondrial function, DM1 patients’ muscles show mitochondrial accumulation in degenerated myofibrils and disorganization of the sarcoplasmic reticulum [68]. These muscles show reduced Coenzyme Q10 (CoQ10) levels, a component of the electron transport chain that participates in aerobic cellular respiration [69]. Blood samples confirmed an inverse correlation between CoQ10 levels and CTG expansion length in DM patients [70]. Moreover, DM1 fibroblasts have an impaired ATP production by misregulation of mitochondrial oxidative phosphorylation system and altered mitochondrial dynamics [71].

At the molecular level of metabolic defects, hyperphosphorylation of CUGBP, which is a common alteration of DM1, leads to abnormal splicing of the insulin receptor (IR) mRNA, lacking exon 11 [72]. The immature form that lacks exon 11 is mainly expressed in embryonic tissues and shows high affinity to IGF-II. On the other hand, the mature form is expressed in adult tissues and binds to insulin. As a result, due to the abnormal splicing, DM1 skeletal muscles are characterized by a predominant expression of the immature isoform, which leads to insulin insensitivity [73] and to a lower insulin signaling activation [74]. This splicing defect seems to be independent of muscle fiber type, as both fiber types show a reduced expression of the adult IR isoform [75].

In summary DM1, present multiple cellular and molecular phenotype that resemble an accelerated aging process. Indeed, the accumulation of clinical and molecular features facilitated the hypothesis that DM1 is a model of progeria, at least, with accelerated aging.

## 4. Metformin

Metformin has its origin in the herb *Galega officinalis*, which was used for centuries to treat many ailments including polyuria [76]. In 1922, metformin (dimethyl biguanide) was synthesized by Werner and Bell, and a few years later it was demonstrated that had the potential to decrease blood glucose with fewer gastrointestinal adverse effects than others therapies used for the same purpose [77]. Today, metformin is one of the most prescribed drugs worldwide and it has become a first-line therapy for the treatment of type 2 diabetes (T2D) as a monotherapy or in combination [78].

Metformin inhibits mitochondrial complex I (NADH:ubiquinone oxidoreductase) and elicits the phosphorylation and activation of AMP-activated protein kinase (AMPK), that can produce diverse pharmacologic effects such as the inhibition of glucose and lipid synthesis [79,80,81]. In this sense, the administration of metformin decreases intestinal glucose absorption, improves peripheral glucose uptake and utilization, lowers fasting plasma insulin levels and increases insulin sensitivity, which result in a reduction in blood glucose concentration [82]. Sixty years of clinical experience and trial data have yielded almost no safety concerns for metformin. The major exception is that metformin causes subclinical increases in lactic acid and appears to cause lactic acidosis in extreme overdose [83]. Over the years, different studies revealed additional potential indications of metformin. Thus, it can also be used as a cardiovascular protective agent [84], a neuroprotective agent [85], it has a role in autoimmune diseases [86], and as an anticancer agent [82].

### 4.1. Metformin Targeting Aging: At Molecular Level

The molecular mechanisms of metformin action are extensive and seem to attenuate several hallmarks of aging [87,88].

Indeed, studies in multiple in vivo models and cell lines have elucidated the role of metformin in targeting fundamental molecular pathways and processes of biological aging describing that metformin exerts potent anti-aging effects [87,89,90]. In this sense, metformin extended the lifespan of mice [91,92] and in a *C. elegans* aging model [93], as well as the survival time in age-related diseases [94]. Metformin attenuates specially four of the hallmarks of aging and acts directly on key molecular players of several pathways. Thus, metformin modulates nutrient-sensing pathways that are critical for the maintenance of energy homeostasis [95,96], suppresses inflammation [97], reduces oxidative stress and DNA damage [98] and inhibits protein synthesis via mTOR pathway and rescues protein misfolding [99]. Because all hallmarks of aging are highly interconnected, the regulation of any of them by metformin, has consequences for systemic attenuation of other hallmarks. Different authors suggest that the action of metformin on those hallmarks has effects on mitochondrial function [81], DNA and histone modifications [100], in prevention of telomere shortening and lowering senescent cell burden via downregulation of the Senescence Associated Secretory Phenotype [97]. In addition, metformin induces stem cell rejuvenation capacity and delays stem cell aging [101]. All these benefits at molecular and cellular level are corroborated by the fact that metformin extends lifespan, healthspan and delays aging in several animal models [91,92,102].

### 4.2. Metformin Targeting Aging: At Clinical Level

Epidemiological studies have further confirmed the metformin’s gerotherapeutic effect with the decrease in the incidence of multiple age-related diseases, in both diabetics and non-diabetics adults, and in an independent manner of its therapeutic efficacy as an anti-diabetic drug [103,104]. Diabetics that take metformin displayed reduced mortality and increased lifespan compared to non-diabetics [88,105]. Emerging evidence suggests that metformin may preserve cognitive function as well. Thus, observational studies of metformin-treated T2D patients reported lower rates of dementia [106], reduced depressive symptoms [107] and reduced the risk of cognitive impairment, with the lowest risk seen in those patients with longer-term (>6 years) metformin use [108]. Moreover, metformin targets age-associated metabolic and non-metabolic pathways in the skeletal muscle of older human adults [109]. In addition, association studies showed that metformin diminishes the incidence of age-related cancers by 31% [109,110]. This latter result is also supported by multiple preclinical studies performed both in vitro and in vivo models confirming metformin’s role in attenuating tumorigenesis. The mechanisms for all these activities are quite broad and include to reduced insulin levels, improved insulin action, decreased IGF-1 signaling, and activation of AMPK [82].

These advancements have been the proof-of-concept to initiate clinical trials to investigate the efficacy of interventions in targeting human aging, starting with the TAME (Targeting Aging with MEtformin) study [111,112]. The TAME study, announced in 2017, aims to prove the concept that human aging can be targeted, in this case with metformin, and to push the FDA to approve “aging” as a target for drug discovery and development. Indeed, metformin became the first molecule with FDA permit to perform a clinical trial directly targeting aging [111,113].

In conclusion, metformin exerts beneficial effects on aging, longevity and healthspan in several preclinical models and it is effective and powerful to target several age-related processes and diseases in humans.

### 4.3. Metformin Targeting Myotonic Dystrophy

As stated above, alterations in the insulin signaling is a common feature in patients with DM1 and T2D is approximately three times higher in DM1 patients than in matched controls [38,114]. Since metformin is the first-line antidiabetic treatment for this patients [38,115], there have been few studies testing it in DM1 patients and preclinical models. Indeed, different studies in DM1 revealed a beneficial effect of metformin on cellular and molecular mechanisms that are involved in the progression of DM1 (Table 1). In particular, recent evidence suggests that metformin: (1) corrects DM1-related alternative splicing defects; (2) alleviates several age-related molecular alterations; (3) reduces the risk of developing cancer and (4) improves mobility in DM1 patients. Notably, no serious adverse effects were reported in DM1 patients treated with metformin being the most common side effects of metformin, hypoglycemia or lactic acidosis, not reported in DM1 patients [116]. In the next sections, we will detail the effects of metformin in DM1 disease at preclinical and clinical level.

#### 4.3.1. Metformin in DM1 at Preclinical Level

Our group recently studied the role of metformin in several hallmarks of aging using primary fibroblasts derived from DM1 patients as an experimental model. DM1 derived primary fibroblasts showed impaired metabolism and mitochondrial dysfunction, resulting in the production of lower levels of ATP and increased reactive oxygen species compared to fibroblasts from healthy donors. Interestingly, treatment with metformin resulted in the restoration of these defects extending the positive effects of metformin to mitochondrial activity. Treatment with metformin for 72 h was as well able to significantly improve the impaired cell proliferation of DM1 fibroblasts measured by cell viability, Ki67 and phospho Histone 3 [71]. Following with in vitro human models, Laustriat et al. explored the impact of metformin on DM1-related splicing events in mesodermal precursor cells (MPCs) derived from human embryonic stem cells and in primary myoblasts derived from patients and healthy controls. First, they found that treatments with metformin during 48 h did not affect viability, cytotoxicity, or apoptosis up to doses of 35 mmol/l and tended to reduce proliferation. Interestingly, the administration of metformin promoted a corrective effect on several splicing defects associated with the disease including the restoration of *INSR* exon 11 splicing defects. Indeed, deep RNA sequencing revealed 1171 genes regulated by metformin in DM1 MPCs. Biological processes corresponding to cell cycle, response to DNA damage, cytoskeleton and ATP binding were enriched. Moreover, 89 common splicing events were deregulated. Gene set enrichment analysis of the splicing events regulated by metformin identified a set of genes involved in cytoskeleton, nuclear lumen, RNA binding, or with kinase activity. The biological effects of metformin were shown to be compatible with therapeutic dosages in a clinical investigation involving diabetic patients. Thus, metformin triggered *INSR* exon 11 inclusion was also rescued in peripheral blood lymphocytes. Thus, the drug could act as a modifier of alternative splicing of a subset of genes. Metformin has different mechanisms that targets the splicing machinery in DM1: via AMPK-dependent and independent pathways and through the downregulation of RBM3 RNA-binding protein and could be in part defined by the overlap between the targets of RBM3 and those of MBNL1 [117].

The impact of metformin on alternative splicing was further tested in a mouse model of the disease. In this case, HSA^LR^ mice treated with metformin for 10 days failed to recover mis-splicing of DM1-affected genes and myotonia [118], maybe due to a limited activation of AMPK. However, a direct AMPK activator similar to metformin, 5-aminoimidazole-4-carboxamide riboside (AICAR), improved muscle relaxation and muscle histology in HSA^LR^ mice with the partial correction of chloride voltage-gated channel 1 (*CLCN1*) mis-splicing, reduction in RNA foci and decreased aggregation of toxic CUGexp mRNA [118,119]. Future studies of additional models should be performed to confirm the effect of metformin on cell activities and as a modifier of splicing machinery. Moreover, additional DM1 animal models and in vivo phenotypes should be characterized in order to further test the impact of metformin in DM1 preclinical models. In this direction, DMSXL mice carrying >1000 CTG repeats displays high mortality, growth retardation, and muscle defects and treatment with metformin functional benefit on motor testing [116,120].

#### 4.3.2. Metformin in DM1 at Clinical Level

As stated above, metformin was tested in DM1 diabetic patients and shown to restore some alternative splicing events in peripheral blood lymphocytes [117]. Increased evidence confirms that DM1 patient’s present increased risk for cancer development and metformin exerts a potent anti-tumorigenic effect. Alsaggaf et al. assessed the relationship between T2D, metformin and the risk of developing cancer in DM1 patients. The authors studied a cohort of 913 DM1 patients and an age-, sex- and clinic-matched cohort of 12,318 DM1-free controls finding that DM1 patients with T2D displayed a higher risk to develop cancer, compared to those without T2D [114]. As expected, T2D was found to be more prevalent in DM1 patients than in controls (8% vs. 3%, *p* < 0.0001) [114]. Importantly, cancer risk was not elevated in DM1 patients with T2D taking metformin, whereas no significant associations were found between T2D and cancer risk in healthy controls either users or nonusers of metformin [114]. These results show the potential role of metformin in the prevention of DM1-related cancer development. The biologic mechanisms underlying metformin’s chemopreventive effects are not understood. We hypothesize that could include direct mechanisms through AMPK-dependent and AMPK-independent pathways and indirect mechanisms through modifications of blood glucose and insulin levels, which could influence the survival of cancer cells. Insulin and insulin-like growth factor 1 (IGF-1) can promote tumorigenesis by stimulating the proliferation of epithelial cells. Thus, decreasing the insulin level may prevent neoplastic activity [121]. Metformin can also affect the inflammatory processes that are reported to play a significant role in tumor progression and in DM1 patients [122].

**Figure 1:**
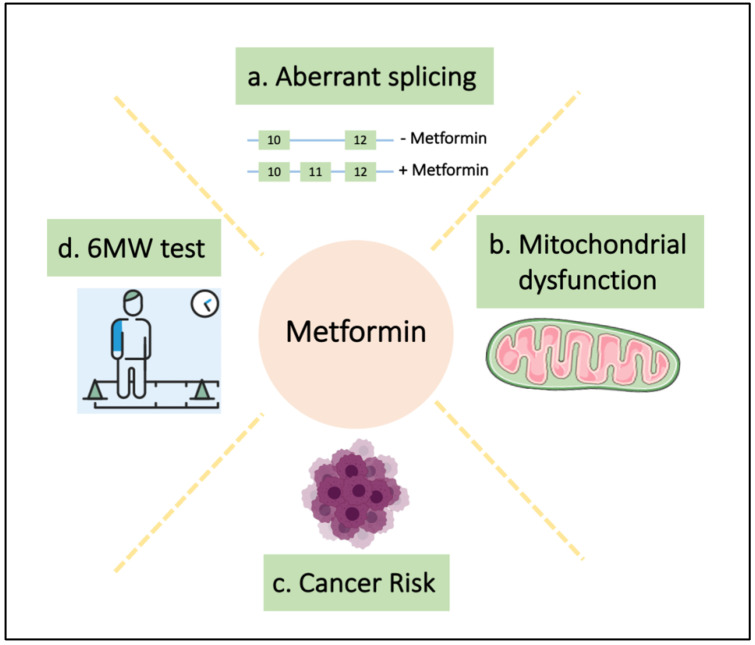
Metformin restores multiple phenotypes in DM1. (**a**) Loss of exon 11 in *INSR* gene is associated with insulin resistance in DM1 patients and metformin restores the inclusion of this exon [117]; (**b**) metformin treatment restores the impaired mitochondrial metabolism of DM1 fibroblasts [71]; (**c**) DM1 patients have an increased cancer risk and metformin reduces this risk in DM1-T2D patients [114], and (**d**) finally, metformin improves the 6-min walk test in a clinical trial [116].

Extending the possible benefits and use of metformin in DM1 patients, a small clinical trial explored the effects of metformin administration on mobility in non-diabetic DM1 patients, [116]. It was a 52-week monocentric, randomized placebo-controlled double-blind phase II study in which oral metformin or placebo was provided three times daily, with a dose-escalation period over 4 weeks up to 3 g/day, followed by 48 weeks at maximum dose. The primary outcome was the change in the distance walked during the 6-min walk test (6MWT), but also other measures regarding functional capacity were assessed. The 6MWT was selected because it has been widely used over the years and facilitates a standard comparison of results [123]. Similar results were obtained in all physical measures and in the mean 6-min walk test at baseline. However, for the 23/40 patients who completed the 1-year study, statistically significant differences between the groups were observed, with the treated group (n = 9) improving 6MWT in 29 m compared to the placebo group (n = 14). Moreover, there was a statistically significant improvement in total mechanical power during gait although; metformin did not seem to have visible effects either on myotonia or on muscle strength. Within that general framework, these encouraging results support the putative role of metformin in the treatment of myotonic dystrophy patients. In this direction, a replication study in a multicenter Phase III clinical trial (2018-000692-32) is currently ongoing in Italy with approximately 100 DM1 patients who will receive metformin for 24 months. The results of this clinical trial are expected in the next year and will provide new evidence for the use of metformin in DM1. In summary, these studies reveal the efficacy of metformin delaying and/or limiting DM1, not only in diabetes but also in additional characteristics of its pathobiology (Figure 1).

### 4.4. Metformin Targeting Other Trinucleotide Diseases

Trinucleotide repeat disorders consist of a group of human diseases, which are a result of an abnormal expansion of repetitive sequences and primarily affect the nervous system.

Metformin has been studied in other neurological diseases caused by microsatellite repeat expansions attenuating, in some cases, common features to DM1 patients. Recently, it has been described in *C9orf72*ALS/FTD mice (GGGGCC, *C9Orf72* gene), metformin inhibits PKR phosphorylation and activation and decreases repeat-associated non-ATG translation (RAN) protein levels [124]. Previously, it has been described that RAN translation occurs in DM1 cells when CAG expansion constructs are transcribed in the absence of an ATG start codon and produces toxic polypeptides [125]. In addition, metformin has shown beneficial effects in other repeat expansion diseases. Huntington (CAG repeats, *HTT* gene) disease mice improve with metformin [94,126], and Huntington disease patients that take metformin have been shown to score better on cognitive tests [127]. In addition, metformin restores most of the phenotypic defects observed in Fragile X syndrome (CGG repeats, *FMR1* gene) mouse model [128] and X-fragile patients treated with metformin improve in language development and behavior [129].

## 5. Concluding Remarks

DM1 is a disease without an approved therapy to slow or stop the progression of the disease. Supportive treatments, preventative measures and clinical surveillance are the only options available for DM1 patients [130]. In recent years, several novel compounds and strategies have been proposed as anti-DM1 therapy [130]. They have mostly been focused on targeting CUG repeats and DMPK via reducing its expression and/or preventing interactions with MBNL1. However, it remains to be seen if any of these strategies are safe and effective in DM1 patients. Therefore, it is necessary to develop new compounds or strategies with potential therapeutic use in this disease. In this sense, metformin is an interesting compound with no serious adverse effects reported in DM1 patients, with promising preclinical and clinical results and an ongoing phase III clinical trial.

Metformin is a drug that has been used for more than 50 years to combat T2D and is known by clinicians for its great effectiveness against T2D and for the few adverse effects. Indeed, the most common side effects of metformin, hypoglycemia or lactic acidosis, were not reported in DM1 patients [116]. Several groups independently studied the impact of metformin on DM1, discovering that it rescues multiple symptoms related to T2D in DM1 patients [115]. Importantly, metformin exerts benefits outside of the management of T2D, since it rescues alternative splicing defects in DM1 samples in vitro and in vivo [117], restores molecular and cellular phenotypes in vitro [71], it is able to reduce the risk of developing cancer of DM1 patients [114], as well as increases the functionality and mobility of patients with DM1 [116]. Moreover, these extensive effects might be the consequence of the antiaging effects that metformin exerts [88,89]. Several groups propose DM1 as a disease that exhibits, both at the clinical and biological level, a premature aging phenotype [54,55]. Thus, treatment with metformin could have additional benefits for DM1 patients, which will be interesting to explore in the future.

## Figures and Tables

**Table 1:** Main findings of the effect of metformin on DM1.

Year	Finding	Reference
2005	The utility of metformin for the management of hyperglycemia in DM1	[115]
2015	Metformin as a modifier of DM1-associated alternative splicing in vitro and in DM1 patients	[117]
2017	Limited effect of metformin treatment in HSA^LR^ mice may be due to a limited activation of AMPK	[118]
2018	Phase II clinical study showing that treatment with metformin for one year in DM1 patients improves the walking distance over 6 min	[116]
2020	Metformin restored impaired cell metabolism, mitochondrial dysfunction and cell proliferation in DM1-derived fibroblasts	[71]
2020	DM1 patients with T2D and using metformin have a lower risk of developing cancer	[114]
Not published	Phase III clinical trial to study the efficacy of metformin on motility and strength in DM1 patients for 24 months	

## Data Availability

Not applicable.
